# Where Does Evidence from New Trials for Schizophrenia Fit with the Existing Evidence: A Case of the Emperor's New Clothes?

**DOI:** 10.1155/2012/625738

**Published:** 2012-04-08

**Authors:** Mahesh Jayaram, Ranganath D. Rattehalli, Clive E. Adams

**Affiliations:** ^1^Leeds and York Partnerships NHS Foundation Trust, Aire Court Community Unit, Lingwell Grove, Leeds LS10 4BS, UK; ^2^Leeds and York Partnerships NHS Foundation Trust, The Newsam Centre, Seacroft Hospital Site, York Road, Leeds LS14 6WB, UK; ^3^Psychiatry, The University of Nottingham Innovation Park, Room B9, Gateway Building, Triumph Road, Nottingham NG7 2TU, UK

## Abstract

Advent of “atypical” antipsychotics has spawned new trials in the recent years and the number of such trial reports has been increasing exponentially. As clinicians we have been led to believe that “atypicals” are better than “typicals” despite the odd dissenting voice in academic and clinical circles. This has been largely ignored until the publication of two landmark, independent, pragmatic trials, Clinical Antipsychotic Trials of Intervention Effectiveness (CATIE) and Cost Utility of the Latest Antipsychotic Drugs in Schizophrenia Study (CUtLASS), which proved that thoughtfully chosen “typical” antipsychotics were as good as the newer “atypicals.” We pooled “leaving the study early data” from Cochrane Reviews that existed before CATIE and CUtLASS and added data from CATIE and CUtLASS to the pool for a “before and after” comparison. Addition of CATIE and CUtLASS data only led to narrowing of the already existing confidence intervals, merely increasing precision, and decreasing the risk of Type II error. Perhaps surprisingly, CATIE and CUtLASS when pooled with the already existing data showed us that we had chosen to turn a blind eye to findings that already existed. This leads clinicians to question as to whether, in future, we need to feel less guilty about crying out early on that the emperor has no clothes on.

## 1. Introduction

Once upon a time (60 years ago) there were almost no pharmacological managements for people with schizophrenia. Although ECT and other physical treatments had been tried including the use of insulin, reserpine, Phenobarbital, and many other agents, nothing was truly successful until chlorpromazine came to use in 1952. The change that this drug ushered in the treatment of people with schizophrenia worldwide was truly revolutionary and formed a watershed that is yet to be surpassed [1]. The advent of chlorpromazine was quickly followed by haloperidol and a whole swathe of other antipsychotics which were often advertised as being equally clinically effective but with different side effect profiles. This seemed to be the case [2, 3], and depot formulations soon followed which represented a further advance in means of administration to a group of people with variable compliance. These developments did not replicate chlorpromazine's initial revolution.

In the early 1960s, psychiatry, always wracked with self-doubt, broadly welcomed the randomised controlled trial as a means of objective evaluation and assuaging doubt [4]. However, the trial-based evidence of the 1960s and 1970s is of variable quality and limited perspective. Most trials were short, small, involved in patients only, and measured outcomes not used in routine care [4]. It is, however, easy to judge the past by standards of today. The first CONSORT statement exhorting researchers to better reporting of trials was only agreed in 1996 [5]. Nevertheless, in recent objective summaries of all trial-based evidence of the only antipsychotics to appear on the WHO list of essential drugs, there is compelling evidence of the short-term benefit of chlorpromazine, haloperidol, and fluphenazine as regards delusions, hallucinations, and thought disorder [2, 6, 7]. There is equally compelling evidence of the considerable adverse effects of, especially, the latter two. Long-term data for these drugs, for an illness that is often life long, are remarkably few. There is no persuasive evidence that these or other drug treatments really have any effect on the negative symptoms of schizophrenia (amotivation, poverty of thought, and avolition) despite advertising to the contrary.

In the 1970s drug patents were running out, and “along came the emperor.” The initial justified wave of enthusiasm for the use of antipsychotic medication gave way to the realism of recognition of partial response to medications. A rediscovery of a better understanding of the truly damaging nature of schizophrenia [8] led to hope that a new, pharmacologically based revolution in the treatment of people with schizophrenia could be coming. Clozapine was the vanguard of a new generation of drugs. It was first formulated in the 1960s, began to be used as an antipsychotic in the 1970s, was withdrawn in most countries because of blood dyscrasias (1978), but was safely reintroduced with blood monitoring in the late 1980s [9]. Clozapine remains a compound with an intriguing effect profile and has a superior efficacy which other drugs lack [10, 11]. It did, however, herald a parade of new compounds. These were often favourably compared to one of the most toxic of the older generation drugs, advertised as equally clinically effective (as older drugs) but with different adverse effect profiles. The idea of the use of these expensive compounds was successfully sold to a receptive population of clinicians, policy makers, and the public. The new drugs, the new Emperors, are moderately effective for this most difficult of illnesses. For some years, however, there have been murmurings in the crowd that the Emperor is in fact not so well clad, although those who have thought the new drugs to be useful additions but not a revolution have been in danger of being accused of heresy. Certainly, recently, researchers have been illustrating how industry sponsorship predicts results [12, 13], and leaders in psychiatry have been stating how, perhaps, the profession has been “beguiled” by industry [14].

There have been calls both for more pragmatic trials to clarify the issue of efficacy of antipsychotic medications [4] and for studies with more independent funding [12, 13]. Two recent landmark independently funded semipragmatic trials, CATIE [15] and CUtLASS [16], addressed issues of antipsychotic discontinuation along with efficacy and adverse effects. Since these studies there have been increasing calls that the emperor is less well-clothed than previously thought [14]. Now, with these two important studies, there is an opportunity to investigate if his attire has been immodest or not.

## 2. Aim

 The paper aims to evaluate the difference made by CATIE and CUtLASS to the already existing evidence on the antipsychotic treatment of schizophrenia using a before and after study design.

## 3. Material and Methods

**Figure 1:**
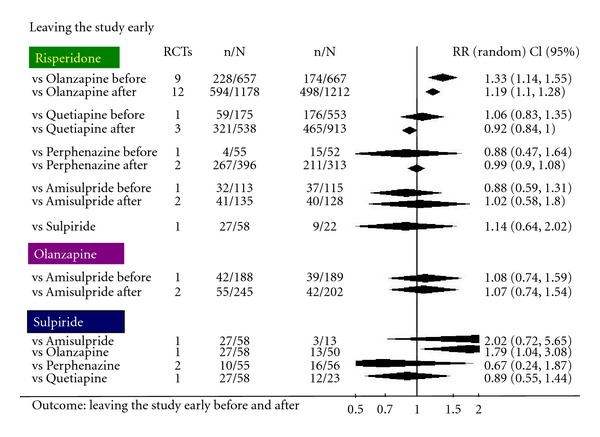
Forrest plot showing comparison of cumulative risk ratios for the outcome of “leaving the study early” before and after CATIE and CUtLASS.

We extracted data on the primary outcome chosen by CATIE (leaving the study early) from Cochrane Reviews relevant to the comparison drugs [17–23] in both CATIE and CUtLASS and undertook a before and after (CATIE and CUtLASS) comparison for the antipsychotics listed in Figure 1. There were other FGAs (first-generation antipsychotics) in CUtLASS, but we did not add them to our comparisons as the numbers in these arms were too small to make any sense of the before-after comparison. Of all the FGAs evaluated in CUtLASS, sulpiride was the most chosen by unblinded clinicians and patients, and we are aware that sulpiride is not available in the US.

## 4. Results and Discussion

What is notable in these results is that, for this particular outcome (leaving the study early), data from CATIE and CUtLASS only increased precision and decreased the risk of Type II error. This was the case in all comparisons except risperidone versus amisulpride where there the precision decreased slightly with the addition of CATIE and CUtLASS. However, in no case did they materially change the impression already available from existing evidence (Figure 1). The only place where this increase in precision seems really powerful is in the comparison including perphenazine. This is because of perphenazine being an old antipsychotic which has been largely ignored by the research community, and the addition of CATIE's data hugely increases the precision of the result. Only CUtLASS compared sulpiride with newer drugs (amisulpride, olanzapine, quetiapine, and risperidone) and found no clear differences. In summary, CATIE and CUtLASS have served only to highlight the findings that were already known by merely increasing precision and reducing the chance of Type II error. At least in terms of the outcome “leaving the study early.”, there is little difference between the new atypical drugs. These two new trials also highlight that, in terms of positive and negative effects, there is little to choose between the newer drugs and intelligent use of older antipsychotics which is consistent with the findings of World Psychiatric Association position statement on antipsychotic treatment of schizophrenia [24], the EUFEST data [25] and the results of systematic overview and metaregression analysis by Geddes et al. [26]. Also meta-analysis by Leucht et al. has potentially contributed to a more balanced view on the differences between first- and second-generation antipsychotics [27, 28].

### 4.1. So What Was the Emperor Wearing?

CATIE and CUtLASS, much more innocent of industry funding and its resultant biases, have highlighted that the emperor(s) were not as well clothed as we had thought.

However, there has been no indication that these data have been hidden from the crowd, by, for example, the industry. More worryingly, there has been the cultural blindness of clinicians, researchers, and recipients of care to data that have been visible. It is not that we do not have effective treatments as we do have them though imperfect. We, as a profession, must use these treatments with care, skill, and humanity and take care not to be easily lead by fashion to join the crowd praising the next ill-clad emperor.

## 5. Conclusions

### 5.1. So Now, What Are the Messages for Us?

#### 5.1.1. Be Humble

There is an element of having been beguiled. This is embarrassing. From this psychiatry can learn that no one will stop a complacent specialty deceiving itself.

#### 5.1.2. Be Scientists

There is encouraging evidence that, even with the biases evident in trials supported by the industry, there are still useful data to be found containing important clinical messages.

#### 5.1.3. Be Vigilant

Marketing, in the form of adverts, presentations, or journal reviews, has an honest priority of supporting the needs of shareholders. In the future it is important for our speciality to be more discerning of what is, and is not, marketing.

#### 5.1.4. Be Thoughtful

There remains no alternative but the thoughtful choice of medication for people with schizophrenia. There is the need for the judicious use of best evidence to skillfully balance good effects with the adverse. CATIE and CUtLASS highlight that the list of antipsychotics from which we should be able to choose should be broad, those choosing older drugs should not be derided, and that use of any of these imperfect treatments can be of value.

## Abbreviations

CATIE: Clinical Antipsychotic Trials of Intervention Effectiveness
CUtLASS: Cost Utility of the Latest Antipsychotic Drugs in Schizophrenia Study
CONSORT: Consolidated Standards of Reporting Trials.
